# Performance, gut morphology, and meat characteristics of broilers housed at a high-density pen and provided with fermented *Averrhoa bilimbi* fruit filtrate

**DOI:** 10.5455/javar.2022.i623

**Published:** 2022-09-30

**Authors:** Sugiharto Sugiharto, Turrini Yudiarti, Endang Widiastuti

**Affiliations:** Faculty of Animal and Agricultural Sciences, Universitas Diponegoro, Semarang, Indonesia

**Keywords:** Broiler, fermented fruit filtrate, growth, gut morphology, meat quality, stocking density

## Abstract

**Objective:**

This study assessed the impact of fermented Averrhoa bilimbi fruit filtrate (FF) on growth, gut morphology, and meat traits of high-stocked broilers.

**Materials and Methods:**

A 2 × 2 factorial trial with stocking densities (9 or 18 birds/m^2^) and drinking 2% FF or plain water was conducted using 378 14-day-old broiler chicks. On day 35, samples were obtained and analyzed.

**Results:**

FF improved feed efficiency and income over the feed cost of high-stocked broilers by about 7.63% and 10%, respectively, compared to high-stocked broilers receiving only water. FF decreased duodenal crypt depth and meat water-holding capacity. Meats from high-stocked broilers receiving FF showed lower cholesterol than other meats. Lower cholesterol/high-density lipoprotein (HDL) and higher HDL/low-density lipoprotein were found in meats from broilers receiving FF. Total unsaturated fatty acid (UFA) was higher in meats of high-stocked broilers receiving FF than others. The UFA/saturated fatty acid was lower in meats of high-stocked broilers receiving plain water. High-stocked broilers given FF had higher meat n-3 polyunsaturated fatty acid (PUFA). FF enhanced meat n-6 PUFA levels. The n-3/n-6 PUFA increased with high density and drinking FF.

**Conclusion:**

Drinking FF improved gut morphology and meat qualities of broilers housed in high-density pens. FF may be an excellent alternative to improve the growth and meat qualities of broilers raised in high-density houses.

## Introduction

The broiler house is one of the most costly aspects of a broiler farm. Because broiler houses have such a high investment cost, broiler producers frequently grow birds at a high stocking density to maximize profits while lowering investment costs. In addition to the efficiency benefits, stockings in high-density pens have been linked to slower growth rates, compromised physiological conditions, and broiler well-being [1,2]. Also, high stocking density-induced stress has been associated with short intestinal villi, resulting in broilers’ poor intestinal digestive and absorptive functions [3]. Moreover, decreased carcass weight [2,4], abnormal color, and reduced water-holding capacity (WHC) [5] have been observed in broiler meat stocked in high-density pens.

Given that broiler growth rate and meat quality are a source of concern in the broiler industry, the poor growth performance and meat quality must be ameliorated. Using synthetic antioxidants to combat oxidative stress is prevalent among broiler producers. Synthetic antioxidants could improve broiler performance and meat quality under stressful conditions [6]. On the other hand, long-term use of synthetic antioxidants may leave residue in broiler meats, harming consumers [6,7]. Because of this, it is very important to find natural antioxidants that can be used in a safe way to help reduce stress caused by high stocking densities in broiler production.

Organic acids have been shown to have antioxidant activity, which can help chickens cope with stress [8,9]. Organic acids also improved intestinal ecology and morphology, which thereby helped broilers grow faster and healthier [7,9]. Meat quality treatment with organic acids has been shown to reduce cholesterol and saturated fatty acid (SFA) while enhancing polyunsaturated fatty acid (PUFA) in broiler meats [10]. Similar to organic acids, LAB has been observed to exhibit antioxidant action [11]. Likewise, the lactic acid bacteria (LAB) treatment has improved intestinal digestive and absorptive functioning due to increased intestinal villi height (VH) of broilers [7]. With respect to meat quality, Yulianto et al. [12] have recently discovered that the dietary treatment of *Lactobacillus casei* enhanced high-density lipoprotein (HDL) levels while decreasing low-density lipoprotein (LDL) and cholesterol in broiler meats. Furthermore, the LAB treatment enhanced the levels of n-3 PUFA, including eicosapentaenoic acid and docosahexaenoic acid, in meats. In search of natural sources of organic acids, attention has been paid to acidic (sour-tasting) fruits. *Averrhoa bilimbi* L. is an acidic fruit with a high concentration of organic acids and phenolic substances [13,14]*.*

Regarding LAB sources, fermented products are generally well recognized as rich in LAB [15]. Shrimp paste, made from fermented shrimp, is a natural LAB source [16,17], containing 4–6 log CFU/gm of LAB [16]. *Lactobacillus plantarum* is the most common LAB species found in Indonesian shrimp paste [17].

Previously, Mareta et al. [13] showed that providing fermented *A. bilimbi* fruit filtrate (FF) using Indonesian shrimp paste (as a fermentation starter) improved feed efficiency, physiological parameters, and the intestinal microbial population of broilers. The beneficial impact of the FF was evident in broilers reared in pens with normal density [13]. However, the impact of such treatment, especially on growth, intestinal morphology, carcass criteria, and meat quality, has never been determined on broilers under stress conditions. Given that the elevation in the number of birds raised per square meter is one of the attempts to maximize cage use while minimizing cage investment costs, the application of natural antistress (instead of synthetic antistress) to ameliorate the adverse impact of stress due to high-density pens becomes critical. The current study assessed the effect of providing FF on productive parameters, gut morphology, carcass characteristics, and meat quality in broilers housed in high-density pens. It was hypothesized that giving FF improved production parameters, intestinal morphology, carcass proportions, and meat characteristics of broilers raised under high-density-induced stress conditions.

## Materials and Methods

### Ethical approval

The Animal Ethics Committee of Universitas Diponegoro’s Faculty of Animal and Agricultural Sciences approved the *in vivo* study (No. 57-04/A3/KEP/FPP).

### Production of FF

*A. bilimbi* fruit (ripe condition) was picked from the campus gardens, while shrimp paste was acquired from Rembang Regency, Central Java Province, Indonesia. The conventional plate counting method using de Man Rogosa and Sharpe’s agar (MRS; Merck KGaA, Darmstadt, Germany) cultured anaerobically at 38°C for 48 h, shrimp paste contained 14.15 ± 0.33 log CFU/gm. Mareta et al. [13] described the fermentation of *A. bilimbi* fruit filtrate with shrimp paste. Briefly, *A. bilimbi* fruit was washed and drained before being crushed with an electric blender and filtered through a cheese cloth. The shrimp paste was then inoculated (gm:l) into the prepared fruit filtrate (pH 1.45 ± 0.06, determined with a portable pH meter, OHAUS ST300) in an anaerobic jar and left at room temperature. The FF had LAB counts of 30.37 ± 0.16 log CFU/ml and a pH of 1.30 ± 0.08 after 4 days of incubation. The fermented products were kept at −10°C until utilized in *in vivo* studies.

### Broiler chicken study

Four hundred 1-day-old Lohmann broiler chickens (36.6 ± 0.10 gm; means standard ± deviation) were fed a commercial starting feed comprising 14% water, 20% protein, 5% fiber, 5% total fat, and 8% ash (according to label) until day 14. The birds were individually weighed on day 14, and 378 chicks (average body weight of 370 ± 9.25 gm) were employed in the current investigation. The *in vivo* study was set up in a factorial arrangement with two factors, i.e., stocking density (density of 9 birds/m^2^ or high density of 18 birds/m^2^) and treatment with 2% FF (from drinking water) or none. Within each density group, half of the chicks received FF via drinking water, while the other half received just plain water. As a result, there were four distinct treatment groups, each with seven pens. From day 14, the chickens were fed a mash-based formulated grower diet (Table 1).

Chicks were inoculated with the Newcastle disease vaccine *via* their eyes (day 4) and drinking water (day 18). Drinking water gave the Gumboro vaccine to the chicks on day 12. During the experiment, the chicks were reared in an open-sided house using rice husk matting. During the trial, the birds were kept in a constant light condition.

### Sample collection and analysis

On day 35, each chicken was weighed individually. One male chick from each pen was killed, de-feathered, and dissected on the same day. For small intestine histological assessment, the segments of the small intestine were collected for about 2 cm, and the segments were placed in a 10% neutral formalin buffer. The edible giblets (heart, liver, and gizzard) and abdominal fat were taken and weighed. The commercial proportions of chicken were also assigned. Breast meat (without skin) was acquired to evaluate the proximate composition and physical properties, meat color, fatty acid levels, and cholesterol content. The meat samples were frozen at −10°C until they were analyzed.

**Table 1.** Ingredients and chemical constituents of feed (day 14–35).

Items	(%, unless otherwise noted)
Yellow maize	58.5
Palm oil	3.00
Soybean meal (crude protein of 44.15%)	34.7
DL-methionine, 990 gm	0.19
Bentonite	0.75
Limestone	0.75
Monocalcium phosphate	1.30
Premix^a^	0.34
Chlorine chloride	0.07
Salt	0.40
Chemical constituents:	
ME (kcal/kg)^b^	3,000
Crude protein	20.0
Crude fiber	5.51
Ca	1.02
P (available)	0.58
Feed price (IDR/kg)	7,600

a Provided per kg of feed: 1,100 mg Zn; 1,000 mg Mn; 75 mg Cu; 850 mg Fe; 4 mg Se; 19 mg I; 6 mg Co; 1,225 mg K; 1,225 mg Mg; 1,250,000 IU vitamin A; 250,000 IU vitamin D_3_; 1,350 gm pantothenic acid; 1,875 gm vitamin E; 250 gm vitamin K_3_; 250 gm vitamin B_1_; 750 gm vitamin B_2_; 500 gm vitamin B_6_; 2,500 mg vitamin B_12_; 5,000 gm niacin; 125 gm folic acid; and 2,500 mg biotin.

b Metabolizable energy (ME) was calculated according to the following formula [45]: 40.81 [0.87 (crude protein + 2.25 crude fat + nitrogen‐free extract) + 2.5].

Histopathological measurements of small intestine segments were performed according to Tunc et al. [18]. Hematoxylin and eosin were used to stain 5 µm intestinal slices. The VH and crypt depth (CD) were evaluated with an optical microscope linked to the camera. The gut morphology of each bird was determined based on five measurements. Before analyzing the meat, it was thawed for 30 min at room temperature. To determine the pH, 1 gm of meat was homogenized thoroughly with 9 ml of water, and the pH value of the filtrate was determined with a digital pH meter. The WHC of breast meat was assessed according to press techniques with filter paper [19]. Meat proximate ingredients were measured using the basic proximate analysis [20]. To measure the amount of cooking loss, meat was inserted into a plastic bag and cooked (at 80°C) for an hour. Before weighing, the meat was permitted to cool, and cooking loss was defined based on the weight divergence before and after the meat was cooked. Stewart et al. [21] described a modified saponification technique for measuring cholesterol in meat. After saponification, enzymatic techniques (using an enzyme kit from Merck Diagnostica, Darmstadt, Germany) were used to measure the meat’s cholesterol, LDL, and HDL levels [22]. The levels of meat fatty acids were measured using gas chromatography. The fatty acids were identified based on the comparison of the retention time of each sample with the standard retention time. Fatty acids were quantified by normalizing the area percentage and converting it to mg per 100 gm edible portion with a lipid conversion factor [23]. On Mac OS X, meat color was determined using a digital color meter (set to CIE Lab). The values L* (brightness), a* (redness), and b* (yellowness) are used to represent colors.

### Statistical analysis

The data were examined with SAS (SAS Inst. Inc., Cary, NC) based on the general linear model method and a 2 × 2 factorial set-up. Interactions between the major effects were included in the model, but they were removed when they were not statistically significant. The data are outlined as means, standard deviations, and standard errors. Duncan’s multiple range test was used to distinguish statistically distinct means (*p* < 0.05).

## Results

### Growth and economic performances of broilers

From day 14 to 35, data on broiler growth and economic performance are shown in Table 2. There was no interaction (*p >* 0.05) between treatments on broiler total live weight, slaughter weight, and daily gain. Yet, the pen’s density significantly impacted the above-measured parameters. The total live body weight of broilers per square meter in the high-density group was higher (*p <* 0.05) than in the normal density group. Normal-stocked broilers had a higher slaughter weight and daily gain (*p <* 0.05) than high-stocked broilers. There was a notable interaction between density and FF in terms of feed consumption, feed efficiency, feed cost, and income over feed cost. Birds raised at a high stocking density and administrated with FF had the lowest average daily feed consumption (*p <* 0.05), while birds raised at normal density and receiving FF had the highest (*p <* 0.05) feed intake. The efficiency of feed and income over feed cost improved (*p <* 0.05) after treatment with FF in drinking water, while feed cost per kg live weight gain decreased (*p <* 0.05) in chicks kept at a high density and provided with FF.

### Intestinal morphology of broilers

The histomorphology of the intestine in broilers is shown in Table 3. The interaction between experimental factors was not substantial throughout the small intestine segments in the current investigation. In the duodenum, high stocking density tended to lower VH (*p* = 0.05) and VH/CD (*p* = 0.09). The FF tended (*p* = 0.06) to decrease the duodenal CD of broilers. The treatments did not influence the gut morphology of the jejunum and ileum (*p* > 0.05).

**Table 2.** Growth and economic performances of broiler chickens (day 14–35).

Items	Normal density		High density		SE	*p-*value		
	–	+	–	+		D	FF	D*FF
Total live BW, gm/m^d^	16,584 ± 743^b^	16,927 ± 446^b^	30,796 ± 1,578^a^	31,081 ± 1,684^a^	466	<0.01	0.51	0.95
Slaughter weight, gm/bird	1,843 ± 82.6^a^	1,881 ± 49.5^a^	1,711 ± 87.7^b^	1,727 ± 93.6^b^	30.3	<0.01	0.38	0.72
ADG, gm/bird/day	69.7 ± 3.99^a^	71.5 ± 2.34^a^	64.3 ± 4.15^b^	65.0 ± 4.43^b^	1.44	<0.01	0.38	0.72
ADFI, gm/bird/day	111 ± 3.82^b^	121 ± 8.01^a^	104 ± 2.76^c^	98.2 ± 4.29^d^	1.93	<0.01	0.34	<0.01
Feed efficiency, %	62.8 ± 3.51^ab^	59.3 ± 3.98^b^	61.6 ± 2.93^b^	66.3 ± 4.29^a^	1.40	0.05	0.67	0.01
Feed cost per kg live BWG (IDR)^c^	12,129 ± 673^ab^	12,858 ± 861^a^	12,367 ± 580^a^	11,508 ± 758^b^	274	0.05	0.81	0.01
Income over feed cost (IDR)^d^	13,609 ± 1,340^ab^	12,668 ± 1,350^ab^	12,433 ± 1,206^b^	13,686 ± 1,478^a^	509	0.88	0.76	0.04

BW: body weight, ADG: average daily gain, ADFI: average daily feed intake, BWG: body weight gain, IDR: Indonesian Rupiah (currency), SE: standard error, D: density, FF: fermented *A. bilimbi* fruit filtrate using shrimp paste, D*FF: interaction between stocking density and fermented *A. bilimbi* fruit filtrate, “–”: chicks receiving plain water, “+”: chicks receiving 2% FF of drinking water.

a,b In the same row, means marked by superscript letters differ significantly (*p <* 0.05).

c Values were taken into account at the time of study as the cost of feed consumed to produce per kg live weight gain.

d Total revenue minus total feed cost was used to calculate values at the time of the study.

**Table 3.** Intestinal morphology of broilers.

Items	Normal density		High density		SE	*p-*value		
	–	+	–	+		D	FF	D*FF
Duodenum								
Villi height (µm)	1,307 ± 152	1,295 ± 201	1,148 ± 335	1,037 ± 370	106	0.05	0.56	0.64
Crypt depth (µm)	88.6 ± 19.3	79.4 ± 10.8	87.8 ± 14.9	76.4 ± 8.89	5.30	0.72	0.06	0.84
VH/CD ratio	15.3 ± 3.25	16.4 ± 1.58	13.4 ± 4.59	13.5 ± 4.16	1.35	0.09	0.68	0.69
Jejunum								
Villi height (µm)	850 ± 434	1,035 ± 331	784 ± 180	775 ± 239	118	0.18	0.46	0.42
Crypt depth (µm)	73.0 ± 12.2	72.8 ± 7.30	76.6 ± 6.92	73.9 ± 4.92	3.12	0.45	0.66	0.69
VH/CD ratio	11.3 ± 4.22	14.7 ± 6.21	10.2 ± 1.63	10.6 ± 3.69	1.61	0.12	0.24	0.36
Ileum								
Villi height (µm)	738 ± 247	682 ± 238	670 ± 273	509 ± 146	87.2	0.17	0.22	0.55
Crypt depth (µm)	70.6 ± 16.7	66.3 ± 7.80	60.9 ± 5.08	77.7 ± 41.1	8.57	0.91	0.47	0.23
VH/CD ratio	10.3 ± 1.20	10.6 ± 5.17	11.1 ± 4.49	7.17 ± 2.15	1.38	0.35	0.22	0.14

VH/CD ratio: villi height to crypt depth ratio, SE: standard error, D: density, FF: fermented *A. bilimbi* fruit filtrate using shrimp paste, D*FF: interaction between stocking density and fermented *A. bilimbi* fruit filtrate, “–”: chicks receiving plain water, “+”: chicks receiving 2% FF of drinking water.

### Carcass traits of broilers

Table 4 presents the data on carcass indices, abdominal fat, and edible giblets of broilers. A significant interaction was observed between the two factors regarding the abdominal fat accumulation of broilers. The abdominal fat deposition was less (*p <* 0.05) in normal-stocked broilers receiving FF than in other birds. The treatments, however, had no influence (*p >* 0.05) on carcass traits and edible giblets.

### Chemical and physical traits of broiler meats

The data on the meat’s chemical and physical traits are outlined in Table 5. There was no interaction (*p >* 0.05) between treatments on moisture, crude protein, crude fat, WHC, cooking loss, and pH of broiler breast meat. Stocking density had no (*p >* 0.05) impact, whereas WHC was reduced in broilers receiving FF breast meat. Cooking loss decreased (*p <* 0.05) with high density but was not affected (*p >* 0.05) by drinking FF. High-stocked meats showed higher (*p <* 0.05) pH values than normal-stocked broiler meats. The FF increased (*p <* 0.05) the meat pH. There was a notable interaction (*p <* 0.05) between these two factors in the lightness values of broiler meats, and it was also shown that a high-density pen reduced (*p <* 0.05) broiler meats. The notable interaction between density and drinking FF was not observed regarding broiler meats’ redness and yellowness values. Indeed, housing broilers in density pens reduced redness levels in the meats (*p* > 0.05). Yet, stocking density had no impact (*p >* 0.05) on meat yellowness values.

**Table 4.** Carcass traits, abdominal fat, and edible giblets of broilers.

Items	Normal density		High density		SE	*p-*value		
	–	+	–	+		D	FF	D*FF
Eviscerated carcass (% live BW)	66.1 ± 1.59	64.2 ± 2.57	64.8 ± 1.70	65.1 ± 0.96	0.68	0.80	0.23	0.11
	% eviscerated carcass							
Breast	35.8 ± 4.09	34.9 ± 1.93	32.7 ± 3.15	35.7 ± 2.34	1.13	0.34	0.36	0.09
Wings	10.7 ± 0.63	10.7 ± 1.76	12.2 ± 1.07	10.5 ± 1.21	0.47	0.19	0.10	0.08
Thigh	15.4 ± 1.15	16.3 ± 1.34	16.7 ± 1.56	16.5 ± 1.12	0.49	0.16	0.55	0.26
Drumstick	14.1 ± 1.34	15.1 ± 0.85	15.1 ± 1.38	14.7 ± 0.60	0.42	0.48	0.50	0.12
Back	23.9 ± 4.33	23.0 ± 1.63	23.3 ± 1.09	22.6 ± 2.11	0.98	0.59	0.41	0.92
Abdominal fat	1.87 ± 0.83^a^	0.93 ± 0.72^b^	1.70 ± 0.51^a^	1.77 ± 0.38^a^	0.24	0.17	0.08	0.04
Edible giblets	6.38 ± 0.27	7.06 ± 0.85	7.21 ± 0.59	7.02 ± 0.83	0.26	0.14	0.35	0.10

BW: body weight, SE: standard error, D: density, FF: fermented *A. bilimbi* fruit filtrate using shrimp paste, D*FF: interaction between stocking density and fermented *A. bilimbi* fruit filtrate, “–”: chicks receiving plain water, “+”: chicks receiving 2% FF of drinking water.

a,b In the same row, means marked by superscript letters differ significantly (*p <* 0.05).

**Table 5.** Chemical and physical characteristics of broiler breast meats.

Items	Normal density		High density		SE	*p-*value		
	–	+	–	+		D	FF	D*FF
Moisture (%)	75.9 ± 0.61	75.0 ± 0.77	75.3 ± 0.47	65.4 ± 4.54	3.28	0.13	0.11	0.18
Crude protein (%)	21.8 ± 0.94	22.3 ± 0.87	22.4 ± 0.52	22.1 ± 0.75	0.21	0.23	0.68	0.07
Crude fat (%)	0.94 ± 0.40	0.93 ± 0.27	0.83 ± 0.14	0.85 ± 0.24	0.07	0.19	0.88	0.87
WHC (%)	41.8 ± 1.42^a^	40.1 ± 1.36^b^	41.1 ± 1.01^a^	39.7 ± 0.80^b^	0.31	0.12	<0.01	0.58
Cooking loss (%)	27.4 ± 1.03^a^	27.7 ± 1.26^a^	26.5 ± 0.97^b^	26.2 ± 1.13^b^	0.29	<0.01	0.98	0.30
pH	6.20 ± 0.07^b^^y^	6.26 ± 0.05^b^^x^	6.50 ± 0.06^a^^y^	6.51 ± 0.04^a^^x^	0.02	<0.01	0.03	0.10
L* (lightness)	48.4 ± 3.41^a^	45.9 ± 5.05^b^	35.2 ± 2.81^d^	39.1 ± 4.84^c^	0.75	<0.01	0.35	<0.01
a* (redness)	4.34 ± 4.26^a^	3.00 ± 2.76^a^	0.44 ± 2.48^b^	1.31 ± 2.85^b^	0.59	<0.01	0.63	0.05
b* (yellowness)	5.98 ± 2.62	5.80 ± 1.48	6.33 ± 1.90	5.29 ± 2.29	0.38	0.82	0.09	0.24

WHC: water-holding capacity, SE: standard error, D: density, FF: fermented *A. bilimbi* fruit filtrate using shrimp paste, D*FF: interaction between stocking density and fermented *A. bilimbi* fruit filtrate, “–”: chicks receiving plain water, “+”: chicks receiving 2% FF of drinking water.

a,b,c In the same row, means marked by superscript letters differ significantly (*p <* 0.05).

x,y Means marked by superscript letters differ significantly (*p <* 0.05) between birds receiving plain water and fermented fruit filtrate.

### Cholesterol and fatty acid composition of broiler meats

The cholesterol and fatty acid content of broiler breast meat are outlined in Table 6. Significant interactions between experimental factors were found for cholesterol, HDL, LDL, total unsaturated fatty acids (UFA), UFA/SFA ratio, and n-3 PUFA. However, a substantial interaction was not observed regarding cholesterol to HDL ratio, HDL to LDL ratio, total SFA, n-6 PUFA, and n-3 to n-6 PUFA ratio. Compared with other broiler meats, meats from broilers stocked at high density and receiving fermented fruit filtrate and from broilers stocked at normal density and receiving plain water had lower (*p <* 0.05) cholesterol levels. The meat of chicks kept at normal density and provided with FF had the greatest (*p <* 0.05) HDL and LDL values compared to other meats. In meats from broilers given fermented fruit filtrate, the cholesterol to HDL ratio was lower (*p <* 0.05), while the HDL to LDL ratio was higher (*p <* 0.05) than in meats from broilers given plain drinking water. The total SFA in broiler meat in high-density pens was higher (*p* < 0.05). When comparing meat from high-stocked broilers given fermented fruit filtrate to meat from other broilers, the total UFA was higher (*p <* 0.05). In meats of high-stocked broilers obtaining water, the UFA/SFA ratio was lower (*p <* 0.05) than in other meats. The meat of high-stocked chicks fed FF had the greatest (*p <* 0.05) content of n-3 PUFA. Drinking fermented fruit filtrate enhanced (*p <* 0.05) n-6 PUFA concentrations. In high-stocked and drinking FF, the n-3/n-6 PUFA was higher (*p <* 0.05) than in normal-stocked broiler meats and plain water, respectively.

**Table 6.** Cholesterol and fatty acid profiles of broiler meats.

Items	Normal density		High density		SE	*p-*value		
	–	+	–	+		D	FF	D*FF
Cholesterol (mg/100 gm)	33.0 ± 3.12^b^	48.2 ± 5.20^a^	43.2 ± 3.21^b^	28.8 ± 4.73^b^	1.57	0.01	0.81	<0.01
HDL (mg/100 gm)	7.18 ± 1.12^b^	12.3 ± 1.86^a^	8.88 ± 0.90^b^	9.39 ± 1.11^b^	0.49	0.24	<0.01	<0.01
LDL (mg/100 gm)	9.14 ± 0.45^b^	11.0 ± 2.13^a^	9.33 ± 0.88^bc^	8.37 ± 1.12^cd^	0.49	0.02	0.37	0.01
Cholesterol/HDL	4.68 ± 0.80^a^	4.00 ± 0.70^b^	4.92 ± 0.71^a^	3.13 ± 0.81^b^	0.29	0.29	<0.01	0.07
HDL/LDL	0.78 ± 0.09^b^	1.15 ± 0.26^a^	0.96 ± 0.13^b^	1.14 ± 0.22^a^	0.07	0.23	<0.01	0.22
Total SFA (gm/100 gm)	0.46 ± 0.04^b^	0.49 ± 0.05^b^	0.63 ± 0.21^a^	0.59 ± 0.05^a^	0.03	<0.01	0.84	0.27
Total UFA (gm/100 gm)	0.78 ± 0.08^b^	0.78 ± 0.04^b^	0.69 ± 0.24^b^	0.98 ± 0.07^a^	0.04	0.15	<0.01	<0.01
UFA/SFA	1.72 ± 0.21^a^	1.61 ± 0.22^a^	1.27 ± 0.56^b^	1.68 ± 0.21^a^	0.09	0.04	0.10	0.01
n-3 PUFA (mg/100 gm)	2.62 ± 1.69^b^	11.1 ± 2.33^b^	4.18 ± 6.56^bc^	24.7 ± 19.1^a^	2.72	0.01	<0.01	0.03
n-6 PUFA (mg/100 gm)	200 ± 29.2^b^	237 ± 26.4^a^	185 ± 75.3^b^	254 ± 18.9^a^	11.6	0.95	<0.01	0.18
n-3/n-6 PUFA	0.01 ± 0.01^by^	0.05 ± 0.01^bx^	0.03 ± 0.03^ay^	0.10 ± 0.07^ax^	0.01	<0.01	<0.01	0.10

HDL: high-density lipoprotein, LDL: low-density lipoprotein, SFA: saturated fatty acids, UFA: unsaturated fatty acids, PUFA: polyunsaturated fatty acids, SE: standard error, D: density, FF: fermented *A. bilimbi* fruit filtrate using shrimp paste, D*FF: interaction between stocking density and fermented *A. bilimbi* fruit filtrate, “–”: chicks receiving plain water, “+”: chicks receiving 2% FF of drinking water.

a,b,c In the same row, means marked by superscript letters differ significantly (*p <* 0.05).

## Discussion

Our current results showed that raising broilers at a high-density pen reduced slaughter weight and the average daily gain of broilers. A similar report was also documented by Li et al. [2] and Rashidi et al*.* [24]. This current study observed a novel finding: administering FF filtrate through drinking water enhanced the feed efficiency of broilers raised in high-density pens. This finding, therefore, suggests that using a natural additive in the form of FF can ameliorate the adverse impact of stress caused by high density on the efficiency of feed use for growth in chickens. It seemed that FF might improve the broiler’s digestive and absorptive functions, resulting in higher nutrient availability for growth. In the latter case, the FF may act as an acidifier and probiotic source, thereby improving intestinal ecology and functions [13]. In modern broiler production, a natural novel additive in the form of FF can therefore be used as an alternative to in-feed antibiotics, which are currently prohibited in most countries around the world. Also, FF can be used as a natural antistress that can reduce the detrimental impact of stress due to its high density on broiler performance. In normal-stocked chicks, the effect of FF on feed efficiency was not as apparent as in high-stocked broilers. The reason for the latter condition was still unclear. The birds in normal-density pens seemed to have more prominent space for activity [25], making the birds allocate higher feed-derived energy for physical activity rather than growth.

Regarding average daily feed intake, the increased physical activity was most likely to elevate energy needs, thus increasing feed intake. Because there are fewer birds per square meter, competition for the feeder may be reduced, resulting in increased feed consumption. In a similar trend with feed efficiency, provision of birds with FF through drinking water was found to lower the feed cost per kg live weight gain of chickens, primarily when the birds were housed in high-density pens. Consequently, the income over feed cost of high-stocked broilers also increased with drinking fermented fruit filtrate. Based on the conditions described above, the use of FF could improve the economic or efficiency of modern broiler farms after the prohibition of in-feed antibiotics and the reduction of synthetic antistress agents in various countries around the world.

In the duodenum, broiler VH and the VH to CD ratio were lower when stocking density was high. In accordance with this, Kridtayopas et al. [3] found that increased stocking density was implicated in a substantial decrease in the VH of the duodenum of broiler chickens. In most cases, the lower VH and VH to CD ratio were usually linked to the broiler’s reduced ability to absorb nutrients [26]. As a result, broiler chicken growth rates may be slowed, as in the present trial. A novel finding was found in the current investigation, in which administration of FF tended to reduce CD of broiler chickens regardless of the stocking density effect. According to Alyileili et al. [26], nutrient absorption is promoted by the shallow CD. Feed efficiency in high-stocked pens was better in broilers provided with FF than in broilers that only received water. This could be attributed to the reduced CD due to the fermented filtrate and improved broiler feed utilization and efficiency. As previously discussed, FF is very likely to act as an acidifier and a probiotic [13] that can protect the intestinal mucosa of broilers from damage during stress conditions. Indeed, damage to the intestinal mucosa can increase the demand for new tissue, resulting in increased tissue turnover and deeper crypts in broiler chickens [26]. The latter condition may have an adverse effect on broiler nutrient absorption.

Our data showed that drinking FF was attributed to the reduced abdominal fat proportion of chickens reared in a normal-density environment. The capability of FF to lower fat content seemed to be ascribed to the acid characteristic of the fermented fruit filtrate, which was able to inhibit *de novo* lipid production [27]. Similarly, the presence of LAB in FF may reduce *de novo* lipid formation in the liver while increasing fatty acid oxidation [28]. These conditions may eventually lower the abdominal fat deposition of broilers. However, the fat-lowering action of the fermented filtrate did not appear to be present in high-stocked broilers. In this study, the fat-lowering impact of fermented fruit filtrate was most likely counteracted by the rising effect of high-density-triggered stress on fat deposition. It should be noted that higher stocking density is linked to reduced broiler motility, resulting in increased abdominal fat accumulation [29]. If you want to stop fat from building up in the abdomens of broilers living in high-density pens, you may need to test higher doses (more than 2%) of fermented fruit filtrate in drinking water.

Regardless of the density impact, findings from our study revealed that WHC was reduced in the breast meat of broilers administrated with FF. Attia et al. [30] formerly documented that dietary supplementation of citric or fumaric acids reduced the WHC of broiler breast meats. Owing to the latter study, the high amount of citric acid in FF [14] was most likely responsible for the decreased WHC of broiler meat in the current investigation. In most cases, lower WHC is linked to meats with more crude fat, lower protein, and lower water content [31]. In the current investigation, the crude fat, protein, and water content of meat did not differ amongst the meats. As a result, the lower WHC in the meats of broilers administrated with FF in the current investigation was challenging to justify. In this study, high stocking density was linked to a decreased cooking loss of broiler breast meat. This finding contrasted with that of Nasr et al. [32], who found that housing chickens at a high density resulted in increased cooking and drip loss of meat. The rationale for the lower cooking loss in high-stocked meats is unknown. Commonly, broiler meat with low pH values is associated with lower WHC and higher cooking loss [31]. In this regard, higher pH values of high-stocked broiler meats may be linked to higher WHC and lower cooking loss than normal-stocked broiler meats. Regarding meat pH, high-stocked broiler meats had higher pH values than normal-stocked broiler meats. Birds raised in normal or low-density pens showed more physical activity than those raised in high-density pens, implying that this may increase the rate of glycolysis [33]. The latter circumstance may result in increased lactic acid production, thereby lowering the pH of broiler meats. In particular, with respect to the effect of FF on the pH values of meats, the treatment has been shown to increase the pH values of meats. An earlier study by Sugiharto et al. [34] pointed out that organic acids (formic acid, butyric acid, or a combination of both) increased the pH values of broiler breast meats. Organic acids in FF most likely reduced postmortem muscle glycolysis, preventing meat pH drop after slaughter [35].

Based on the results of the current experiment, high stocking density was linked to reduced broiler meat lightness. Because there is a negative relationship between pH values and broiler meat lightness, higher pH values in high-stocked broilers are usually related to lower lightness values of broiler meat [31]. The lightness values of meats in this investigation were lower than typical values (ranging from 44 to 53). Meats were frozen before laboratory analysis in this study. The fact that broiler meats have a lower lightness score could be because they were frozen before the test [36].

Regarding the administration of FF, the impact of such an additive did not provide a clear pattern, as fermented fruit filtrate decreased the lightness values of broiler meats under normal density conditions but increased lightness under high-density conditions. Until now, the precise cause of this condition has remained unknown. In the current investigation, stocking broilers in a high-density pen was linked to lower redness levels in the meat. The amount of myoglobin in meats is one of the most critical factors impacting the redness values of meats [31]. Indeed, physical activity is linked to higher myoglobin levels in muscles [37]. Considering this, the lesser activity of broiler chicks in high-density pens may explain the lower myoglobin content and, as a result, lower redness levels of high-stocked broiler meat. In this study, the provision of FF had no impact on the redness and yellowness of meat. It seemed that FF was unable to modify the myoglobin and hence did not affect the redness and yellowness values of broiler meats.

Our finding showed that total cholesterol in meat was higher in high-stocked broilers receiving plain water than in normal-stocked broilers receiving plain water. The stress caused by high density appeared to raise cholesterol levels in broiler meat. Park et al. [38] discovered elevated serum cholesterol levels in ducks kept in high-density pens. Stress-induced increases in corticosterone levels appeared to promote cholesterol production and accumulation in broiler chicken muscle tissues [39]. Indeed, FF could help reduce elevated cholesterol levels in meats caused by high-density stress. The antioxidant properties of *A. bilimbi* fruit [14] were most likely to alleviate stress and, as a result, minimize cholesterol synthesis and accumulation in muscle tissues. The latter assumption, however, should be approached with caution, as the FF increased total cholesterol in meats under normal density conditions. The precise reason for the disparate effects of FF on cholesterol content in broiler meats at normal and high stocking densities remains unknown. However, when the chicks were raised in high stocking density pens, the antistress activity of FF appeared to be more pronounced. The same thing occurred to broiler feed efficiency when they were housed in high stocking density pens (feed efficiency was improved by the additive only when the chicks were raised in high density, but not in normal density pens). Consumers generally prefer meat with lower cholesterol/HDL and higher HDL/LDL since it is less likely to cause atherosclerosis. Promising findings were found in this current trial: the administration of FF reduced cholesterol/HDL and enhanced the HDL/LDL ratio in broiler meats. This condition could be explained by the higher increase in HDL (35.1%) compared to cholesterol (increased by 1.05%) and LDL (increased by 4.87%) levels after consuming FF. In terms of the effect of FF on the cholesterol profile, Pratama et al. [40] discovered that drinking FF (spontaneous fermentation) quadratically increased the serum levels of HDL in chicks. The latter investigators also proposed that the probiotic properties of the FF could modify lipoprotein metabolism, resulting in higher HDL levels. Also, the acid in the fermented fruit filtrate made it easier to digest and use proteins, which led to more lipoprotein (the main part of HDL) being made.

The current experiment showed that stocking at a high-density pen increased total SFA in broiler meat. This result is in line with Simsek et al. [41], revealing that high stocking density is implicated in increased SFA proportions in the breast meat of broilers. In this case, stress due to overcrowding may increase *de novo* fatty acid synthesis, resulting in increased SFA in meats [42]. It was also shown in this study that total UFA was higher in high-stocked broilers receiving FF. The application of such an additive appeared advantageous since customers preferred meats with a higher proportion of UFA, particularly PUFA. According to Simsek et al. [41], rearing broilers at a high density implicated a lower ratio of UFA to SFA in meat in the current investigation.

Interestingly, drinking with FF could prevent broiler meat’s decreased UFA to SFA ratio due to high stocking density rearing. The acids and LAB contents in FF were most likely to ameliorate the stress conditions in broilers, which in turn reduced the liver’s *de novo* fatty acid formation. This inference was supported by Galli et al. [43] and Imran et al. [44], who revealed that acids and LAB-based probiotics reduced the SFA content of broiler meats. In this study, the content of n-3 and n-6 PUFA in meats increased with the provision of FF to the broiler through drinking water. The exact reason for the latter circumstance was not known, but acid content in the FF seemed to be responsible. This is in agreement with Galli et al. [43], who found that the incorporation of acids increased the content of n-3 and n-6 PUFA in broiler meats. It was shown in this current investigation that n-3/n-6 PUFA was higher in high-stocked than in normal-stocked broiler meats. This is in contrast to Simsek et al. [41], who found that under the *ad libitum* feeding regimen, the n-3 and n-6 PUFA ratios rose with a reduced density. The substantially increased content of n-3 PUFA in high-stocked compared to normal-stocked broiler meats appeared to contribute to the higher n-3/n-6 PUFA in high-stocked broiler meats. With regard to FF, the treatment increased the n-3/n-6 PUFA of broiler meat in the present study. Indeed, drinking FF increased both n-3 and n-6 PUFA levels. Yet, the increase in n-3 PUFA was much more pronounced (426%) than in n-6 PUFA (28%). This may explain why broiler meats have more n-3 and n-6 PUFA after drinking FF.

## Conclusion

High stocking density impaired broilers’ production parameters, duodenal morphology, and meat quality. Drinking FF improved gut morphology (decreased duodenal CD) and meat quality (increased meat pH, decreased cholesterol levels and cholesterol to HDL ratio, increased HDL to LDL ratio, UFA, n-3 PUFA, and n-3/n-6 PUFA) of broilers stocked in a high-density pen.

## List of abbreviations

CD: crypt depth, FF: fermented *A. bilimbi* fruit filtrate, HDL: high-density lipoprotein, LAB: lactic acid bacteria, LDL: low-density lipoprotein, SFA: saturated fatty acids, PUFA: polyunsaturated fatty acids, UFA: unsaturated fatty acids, VH: villi height, WHC: water-holding capacity.
